# Zinc Finger and X-Linked Factor (ZFX) Binds to Human SET Transcript 2 Promoter and Transactivates SET Expression

**DOI:** 10.3390/ijms17101737

**Published:** 2016-10-20

**Authors:** Siliang Xu, Ping Duan, Jinping Li, Tristan Senkowski, Fengbiao Guo, Haibin Chen, Alberto Romero, Yugui Cui, Jiayin Liu, Shi-Wen Jiang

**Affiliations:** 1 The State Key Laboratory of Reproductive Medicine, Clinical Center of Reproductive Medicine, First Affiliated Hospital, Nanjing Medical University, Nanjing 210029, China; xu_s@mercer.edu (S.X.); cuiygnj@njmu.edu.cn (Y.C.); 2 Department of Biomedical Science, Mercer University School of Medicine, Savannah, GA 31404, USA; li_j@mercer.edu (J.L.); senkowskitristan@savcds.org (T.S.); guo_f@mercer.edu (F.G.); romero_ah@med.mercer.edu (A.R.); 3 Department of Obstetrics and Gynecology, The Second Affiliated Hospital of Wenzhou Medical University, Wenzhou 325027, China; dppddpp@wmu.edu.cn; 4 Department of Histology and Embryology, Shantou University Medical College, Shantou 515000, China; chenhb@stu.edu.cn

**Keywords:** SET (SE Translocation), I2PP2A (protein phosphatase 2A inhibitor), ZFX (Zinc finger and X-linked transcription factor), transcriptional regulation, gynecologic cancers

## Abstract

SET (SE Translocation) protein carries out multiple functions including those for protein phosphatase 2A (PP2A) inhibition, histone modification, DNA repair, and gene regulation. SET overexpression has been detected in brain neurons of patients suffering Alzheimer’s disease, follicle theca cells of Polycystic Ovary Syndrome (PCOS) patients, and ovarian cancer cells, indicating that SET may play a pathological role for these disorders. SET transcript 2, produced by a specific promoter, represents a major transcript variant in different cell types. In this study, we characterized the transcriptional activation of human SET transcript 2 promoter in HeLa cells. Promoter deletion experiments and co-transfection assays indicated that ZFX, the Zinc finger and X-linked transcription factor, was able to transactivate the SET promoter. A proximal promoter region containing four ZFX-binding sites was found to be critical for the ZFX-mediated transactivation. Mutagenesis study indicated that the ZFX-binding site located the closest to the transcription start site accounted for most of the ZFX-mediated transactivity. Manipulation of ZFX levels by overexpression or siRNA knockdown confirmed the significance and specificity of the ZFX-mediated SET promoter activation. Chromatin immunoprecipitation results verified the binding of ZFX to its cognate sites in the SET promoter. These findings have led to identification of ZFX as an upstream factor regulating *SET* gene expression. More studies are required to define the in vivo significance of this mechanism, and specifically, its implication for several benign and malignant diseases related to SET dysregulation.

## 1. Introduction

SET (SE Translocation), also known as Template Activating Factor-1β (TAF-1β) and protein phosphatase 2A inhibitor (I2PP2A), was originally identified as a part of SET-CAN fusion protein generated by chromosomal translocation in the patient SE with acute undifferentiated leukemia [1]. SET is an evolutionarily conserved gene expressed in many human tissues such as brain and gonadal systems. In the female gonadal system, SET is specifically expressed in oocytes and theca cells [2,3]. In the ovary and testis, SET is highly expressed in the genital ridge of early embryo, which indicates its involvement in gonad development [2].

Studies have shown that SET is a multitasking protein involved in different physiological processes [3,4]. Through physical interaction with protein phosphatase 2A (PP2A), SET inhibits PP2A phosphatase activity, modifies protein phosphorylation status, and regulates multiple cell functions [5,6]. For example, increased SET levels in brain neurons are associated with tau protein hyperphosphorylation, which contributes to the formation of intracellular neurofibrillary tangles, and ultimately, leads to the pathogenesis of Alzheimer’s disease (AD) [7,8]. An increased SET expression and decreased PP2A activity in follicle theca cells of Polycystic Ovary Syndrome (PCOS) patients have been cited to explain the hyperandrogenism in this disease [9,10]. SET can also regulate cell function through PP2A-independent pathways. As a part of the complex inhibiting the histone acetyltransferase, SET may affect histone acetylation modification and chromatin remodeling [11]. Moreover, SET protein is able to specifically recognize the promoters of downstream target genes and serve as a transcription factor [12].

Accumulated data have shown that, through PP2A-dependant and PP2A-independent pathways, SET plays a critical role in the cell cycle regulation and contributes to the development of hematological malignancies and solid tumors, especially gynecologic cancers [13,14]. In breast cancers, SET overexpression is a frequent molecular event associated with shorter overall and disease-free survival of patients [15]. A close correlation between SET expression levels and tumor differentiation was observed in epithelial ovarian cancers [16]. Indeed, two novel anti-SET reagents, OP449 and FTY720, were found to promote cell death induced by cisplatin in ovarian cancer cell lines [17], again pointing to the significance of SET in cancer biology. There is also data indicating that, in cervical cancer HeLa cells, forced SET overexpression and manipulation of SET cytoplasmic localization affected cell motility, implicating SET involvement in cancer metastasis [18].

Taken together, SET dysregulation, most significantly, its overexpression, has been observed in cells/tissues involved in Alzheimer’s disease, PCOS, and cancers. However, the molecular mechanism(s) of *SET* gene regulation is not well understood. Through the use of alternative promoters, *SET* gene produces four mRNA variants with distinct exon 1 of divergent lengths (Figure S1), leading to the synthesis of four SET protein isoforms with different sizes [19]. Among the four transcription variants, transcript 1 (TAF-Iα) and transcript 2 (TAF-Iβ) represent two major transcripts [20]. Studies have shown that transcript 2 is expressed more widely than transcript 1 [2]. Nagata et al. demonstrated that CCRF-CEM, Jurkat, PEER, and NALM-6 cell lines representing early stage hemopoietic lineages only express transcript 2 [2]. In xenopus oocytes and porcine testes, SET transcript 2 is also the major variant participating in the gonad development. Quantitatively, the expression levels of transcript 2 tend to be more stable than those of transcript 1 [2]. Several studies have concentrated on the transcriptional regulation of transcript 1, and found Sp1 and NF-κB to be the major factors regulating transcript 1 promoter activity [19,21,22]. However, the regulation of SET transcript 2 promoter remains to be a knowledge gap. The current study is designed to characterize the transcription regulation of transcript 2 promoter. Identification of the *cis*-elements and binding proteins controlling transcript 2 expression will help us to better understand the SET regulation as a possible mechanism underlying the pathogenesis of aforementioned benign and malignant diseases.

## 2. Results

### 2.1. Identification of the Core Promoter Region Regulating the Expression of SET (SE Translocation) Transcript 2

**Figure 1:**
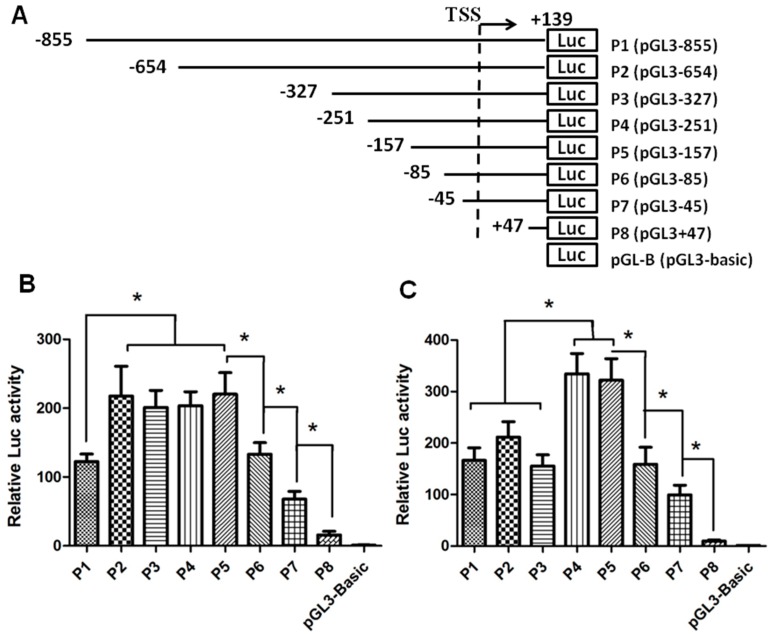
Activities of truncated SET (SE Translocation) transcript 2 promoters in HeLa and HEK 293 cells. (**A**) Schematic presentation of serial deletions in human SET transcript 2 promoter. Promoters of different lengths (P1 to P8) were subcloned into the pGL3-Basic luciferase vector. TSS: Transcription start site; (**B**,**C**) Luciferase activities in transfected HeLa and HEK 293 cells, respectively. Cell transfection was performed with 100 ng of reporter plasmid DNA and 2.5 ng of pRL-TK reference plasmid DNA in 96-well plates. At 24 h post-transfection, firefly and renilla luciferase activities were measured. The firefly luciferase activity was standardized with the correspondent renilla luciferase activity. Data is presented as means ± SD from three independent experiments. (*, *p* < 0.05).

The transcription start site (TSS) and putative promoter were identified with the Promoter 2.0 Prediction Program (Center for Biological Sequence Analysis, Technical University of Denmark, Copenhagen, Denmark; http://www.cbs.dtu.dk/services/Promoter/). Analysis of SET sequences using the SwitchGear Genomics (Carlsbad, CA, USA) pointed to the same TSS as predicted by Promoter 2.0 software. Following PCR amplification, the 994 bp fragment containing the 855 bp promoter and 139 bp 5′ UTR was cloned into the pGL3-Basic luciferase reporter vector. In order to define the proximal sequence essential for the transcriptional control of human *SET* gene, seven sequentially truncated promoters were examined in transfection assay. As shown in Figure 1, the full-length P1 (pGL3-855) generated strong luciferase activities in both HEK 293 and HeLa cells. Interestingly, the truncated promoters displayed different activity patterns in the two cell types. In HeLa cells, deletion to −654 bp from the TSS (P2, pGL3-654) caused a significant increase in promoter activity, indicating the presence of a negative regulatory element(s) in −855/−654 region. In HEK 293 cell lines, however, there appeared to be a suppressive element(s) in −327/−251 region. Thus the *SET* gene transcription might be regulated in a cell-specific manner. On the other hand, in both cell lines, further deletion (P5, pGL3-157; P6, pGL3-85; P7, pGL3-47; and P8, pGL3+47) of the promoter led to a stepwise reduction of promoter activities. A significant reduction was noted to occur between P5 and P8, which corresponds to the region −157/+47. Thus, the 204 bp region harbors strong positive *cis*-active elements indispensable for the promoter activity of the human *SET* gene.

### 2.2. Identification of Crucial Transcription Factors Controlling SET Promoter Activity

**Figure 2:**
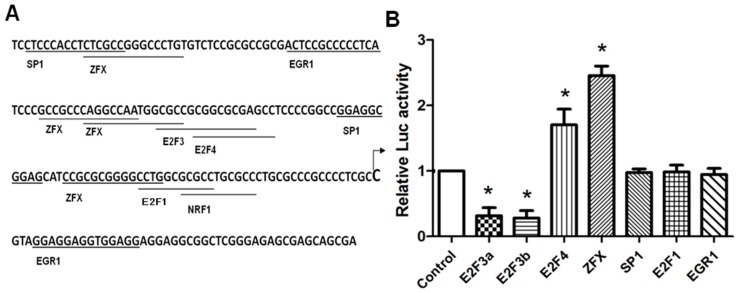
Identification of crucial transcription factors controlling SET transcript 2 expression. (**A**) Positions and sequences of *cis*-elements in the SET core promoter (−157/+47). The transcription start site is indicated by an arrow; (**B**) HeLa cells were co-transfected with 100 ng of P5 plasmid and 100 ng of plasmid expressing transcription factors. At 24 h post-transfection, cells were lysed and luciferase activities were measured. Data is presented as means ± SD from three independent experiments. (*, *p* < 0.05).

The Genomatix software (Munich, Germany; http://genomatix.de/cgi-bin/matinspector_prof/mat_fam.pl.) and JASPAR database (Copenhagen, Denmark; http://jaspar.binf.ku.dk/cgi-bin/jaspar_db.pl.) were used to predict transcription factor binding sites. Several highly scored binding sites were identified within the 204 bp (−157/+47) region, including those for Sp1, E2F1, E2F3, E2F4, EGR1, and ZFX (Figure 2A). To determine whether these putative sites are functional, co-transfection was performed with plasmids expressing these transcription factors and P5 plasmid, the construct containing the minimal promoter. As shown in Figure 2B, E2F1, Sp1 and EGR1 overexpression had no significant effect. E2F3a and E2F3b overexpression significantly suppressed SET promoter activity in HeLa cells (*p* < 0.05). Since E2F3a displayed a similar inhibitory activity in P5, P6, and P7 truncated promoters (Figure S2) The uniform inhibition could be a non-specific effect, or suggest the presence of an E2F3a-related, negative regulatory element further downstream of the deleted region. Since SET is overexpressed in cancer cells, from a pathologic point of view, we are more interested in factors upregulating SET expression, and the E2F3a-mediated negative effects was not pursued further. On the other hand, E2F4 and ZFX overexpression significantly increased SET promoter activity, to 1.8-fold and 2.3-fold (*p* < 0.05), respectively. We elected to focus on ZFX, the factor appeared to account for a large portion of the SET promoter activity.

### 2.3. ZFX Transactivates Human SET Promoter

**Figure 3:**
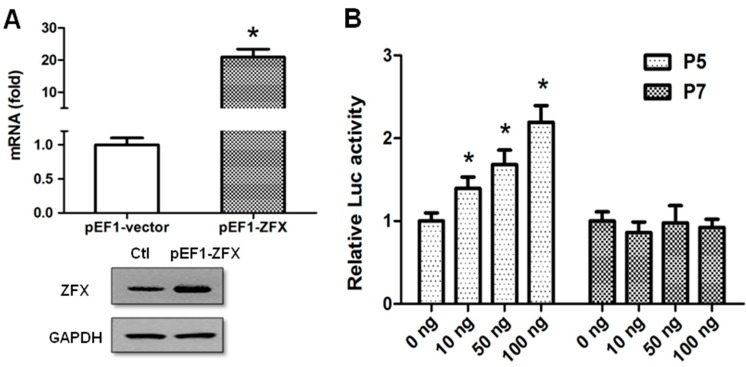
ZFX transactivates human SET transcript 2 promoter. (**A**) HeLa cells were transfected with 100 ng of pEF1-ZFX or pEF1-vector (control). At 48 h post-transfection, total RNA and proteins were isolated and ZFX expression was determined. Top panel: Results of real-time PCR showing a dramatic increase of ZFX RNA mRNA following ZFX overexpression. Bottom panel: Results of Western blotting showing an increased ZFX protein expression. GAPDH protein expression was determined and the results indicated equal protein loading; (**B**) Dose-dependent effect of ZFX on SET promoter activity. HeLa cells were co-transfected with increasing amounts of pEF1-ZFX (0–100 ng, pEF1-vector was used as a “stuffer” to keep a constant DNA amount) and 100 ng of P5 or P7 reporter plasmid. Luciferase activity was measured at 24 h post-transfection. Quantitative data is presented as means ± SD from three independent experiments. (*, *p* < 0.05).

To determine the promoter sequences responsible for ZFX-mediated transactivation, a JASPAR search was performed, and four ZFX-binding sites (−146/−133, −99/−86, −93/−80, and −69/−56) were found to be located between −157/−45, which corresponded to the region deleted from P5 to P7. Therefore, co-transfection was performed with increasing amounts of ZFX-expressing plasmid and P5 (pGL3-157) or P7 (pGL3-45) to determine the significance of these ZFX-binding sites. ZFX overexpression following cell transfection was verified on mRNA and protein levels with the use of real-time PCR and Western blotting, respectively (Figure 3A). While P5 displayed a dose-dependent activation by ZFX overexpression, P7 activity remained stable (Figure 3B). These results demonstrated that ZFX was capable of activating SET promoter, and ZFX sites in the −157/−45 region are likely to play a vital role in the ZFX regulation.

### 2.4. Mutagenesis Study on the Putative ZFX-Binding Sites

**Figure 4:**
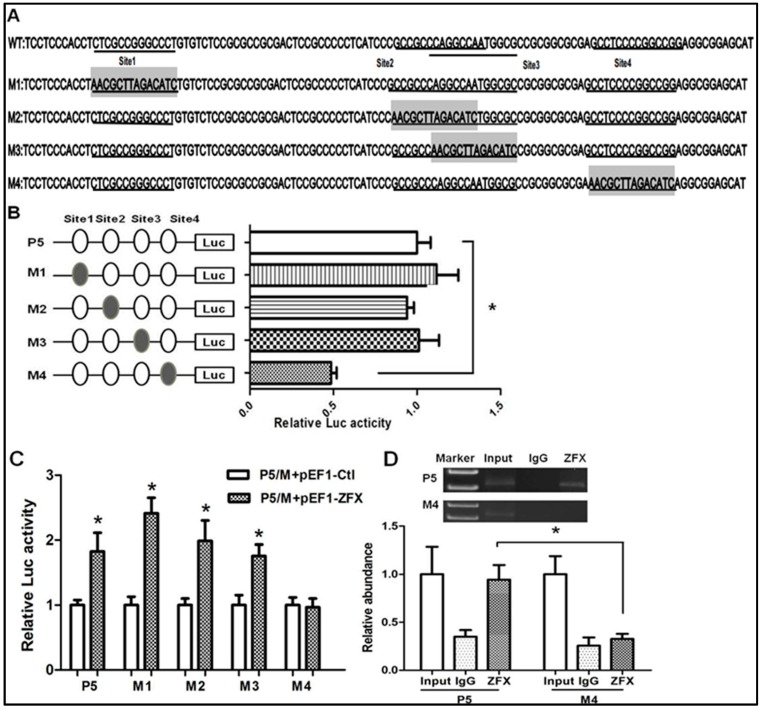
Mutagenesis analysis of putative ZFX-binding sites in SET transcript 2 promoter. (**A**) Locations and sequences of the four ZFX putative binding sites. Note the overlapped Site2 and Site3. Mutated sites, designated as M1–M4, were marked with shadows; (**B**) The left part illustrates the structure of reporter constructs containing mutated sites M1–M4, while the right part shows reporter activities from transfection assays. Reporter activities are expressed as folds of change compared to wild type promoter P5; (**C**) HeLa cells were co-transfected with the wild type P5 or individual mutant promoter constructs and pEF1-ZFX or the pEF1 control vector. Significantly increased reporter activity was found in P5 and M1-3, but not M4 upon ZFX overexpression; (**D**) Transient ChIP assay using cell transfected with either wild type P5 or mutant M4 construct. ZFX-specific antibody was applied as described under Materials and Methods. **Top** panel: A representative image of ChIP results. A clear 322 bp DNA band representing ZFX binding was detected in cells transfected with wild type P5 promoter but not in cells transfected with M4 mutant construct. PCR using DNA templates isolated without immunoprecipitation was used as an input control. PCR using DNA isolated with application of IgG was used as the negative control; **Bottom** panel: Following densitometry analysis, signals of ZFX binding were standardized by those of input, setting the input value as 1. The quantitative data is presented as means ± SD from three independent experiments. (*, *p* < 0.05).

To determine the significance of each ZFX-binding site, site-directed mutagenesis was performed (Figure 4). Four plasmids with each containing a mutation on individual ZFX-binding site were constructed and designated as M1-4 (Figure 4A). The activities of mutant promoters and the parental P5 wild type promoter were compared. As shown in Figure 4B, mutation of the first, second, and third binding sites had no significant effect on promoter activity, whereas mutation of the fourth binding site led to a 50% reduction of promoter activity.

To confirm the above observation, the mutant promoters were examined in the presence of ZFX overexpression. Results of co-transfection experiments with reporters containing mutant promoters and ZFX expression vector indicated that mutation of the fourth site (Site4) led to a blockade of the ZFX-mediated transactivation (Figure 4C). Taken together, these data provided strong evidence for a significant role of the fourth ZFX site in ZFX regulation of SET promoter.

To determine the ZFX binding to its cognate sequences of Site4 (CAGGCCAATGGCGC), we performed a transient chromatin immunoprecipitation (ChIP) assay following cell transfection with P5 or M4 reporter plasmid. As described under Materials and Methods, PCR primers were designed to encompass the immediate upstream of Site4 and downstream of the luciferase-coding sequence. As shown in Figure 4D, ZFX binding was detected in cells transfected with P5 promoter but not in cells transfected with M4. Thus, Site4 (CAGGCCAATGGCGC) is required for ZFX binding and transcriptional regulation of SET promoter.

### 2.5. siRNA-Mediated Knockdown Verified the Significance of Site4

**Figure 5:**
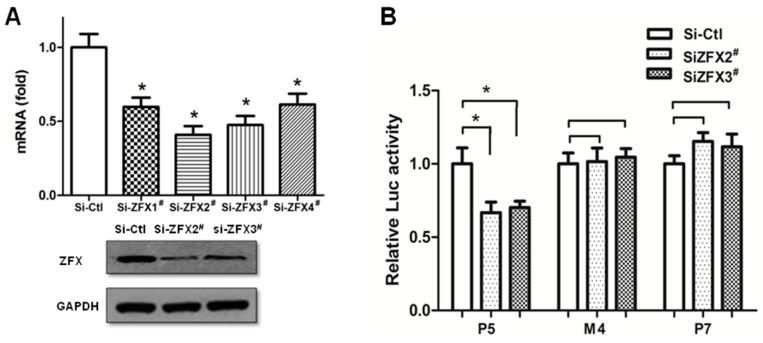
Removal of ZFX-binding site led to a loss of response to ZFX knockdown. (**A**) HeLa cells were transfected with either the non-specific siRNA (Si-Ctl) or one of four ZFX-targeting siRNAs (Si-ZFX1^#^, Si-ZFX2^#^, Si-ZFX3^#^ and Si-ZFX4^#^). Si-ZFX2^#^ and Si-ZFX3^#^ were found to be the most effective for ZFX knockdown. Top panel: Measurement of ZFX mRNA levels. Bottom panel: Western blotting detection of ZFX protein, with GAPDH detected as a control for protein loading; (**B**) When ZFX expression was knocked down, a decreased reporter activity was observed in the P5 construct containing the Site4 ZFX-binding sequences. No change of reporter activity was observed in P7 in which Site4 was deleted. Similarly, no change in reporter activity was observed in M4 in which Site4 sequences were mutated. Results are presented as means ± SD from three independent experiments. (*, *p* < 0.01).

To further confirm the key function of ZFX-binding Site4, endogenous ZFX expression was knocked down with specific siRNAs. Four siRNA sequences were tested for ZFX inhibition. Results of real-time PCR and Western blotting demonstrated that two siRNAs effectively reduced the ZFX expression, with Si-ZFX2^#^ accomplishing 55%–65% reduction of ZFX mRNA and 70%–80% reduction of ZFX protein, and Si-ZFX3^#^ accomplishing 40%–50% knockdown of mRNA and 55%–65% protein (Figure 5A). As shown in Figure 5B, ZFX downregulation by either Si-ZFX2^#^ or Si-ZFX3^#^ readily led to a significant reduction of wild type (P5) promoter activity. However, removal of the ZFX-binding Site4 either by mutation (M4) or deletion (P7) rendered insensitivity to changes of ZFX level. Thus, Site4 appears to play a dominant role for the ZFX-mediated upregulation of SET promoter.

### 2.6. ZFX Binds to Native SET Gene Promoter

**Figure 6:**
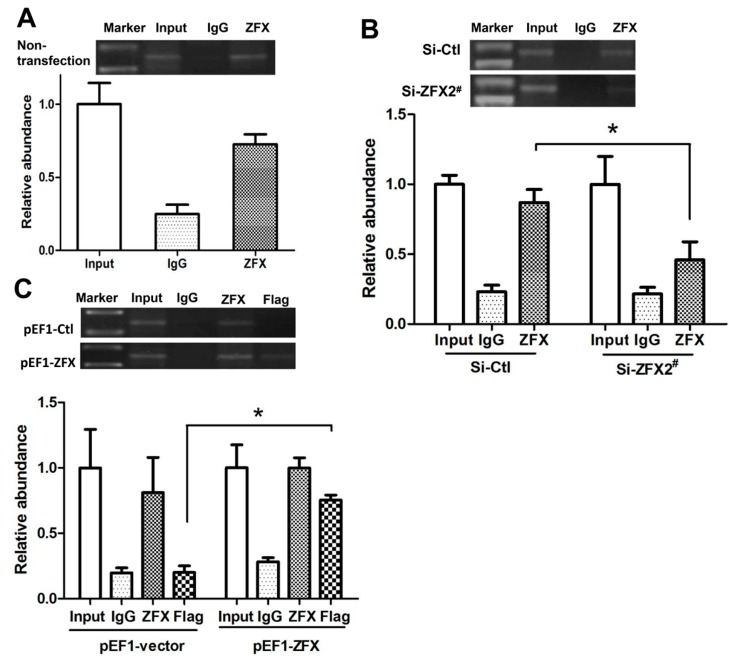
Binding of ZFX to the SET transcript 2 promoter confirmed with ChIP assay. ChIP experiments were conducted using ZFX antibody (2 µg) or Flag antibody (2 µg). In (**A**–**C**), the top panels show representative results of ChIP. The bottom panels present results of densitometry analysis based on three independent experiments. ZFX-binding results were standardized by those of input, setting the input value as 1. Data is presented as means ± SD from three independent experiments. (*, *p* < 0.01). (**A**) When untransfected HeLa cells were used in the ChIP assay, ZFX binding to endogenous SET promoter was readily detected; (**B**) Upon ZFX knockdown using Si-ZFX2^#^, a significant reduction in ZFX binding to endogenous SET promoter was observed; (**C**) ZFX binding to endogenous SET promoter was detected in cells transfected with ZFX-expressing pEF1-ZFX or control pEF1-vector, and Flag antibody detected a strong binding of SET promoter by overexpressed ZFX.

ChIP assays were performed to investigate if ZFX was able to bind to the native SET promoter in HeLa cells. The precipitated DNA was subjected to PCR amplification with primers flanking the genomic region (−147/+26) containing the four ZFX-binding sites. As shown in Figure 6A, ZFX antibodies, but not non-specific IgG, immunoprecipitated the SET promoter DNA, demonstrating ZFX binding to the native SET promoter.

ChIP experiments were also conducted following manipulation of ZFX expression. As demonstrated in Figure 6B, following ZFX knockdown with Si-ZFX2^#^, a decrease in the DNA band representing ZFX binding to SET promoter was observed. In a control experiment, transfection with a scramble siRNA sequence did not significantly affect the DNA band density when compared to the non-transfection group. Correspondently, an increase in the DNA band was observed when ZFX was overexpressed (Figure 6C). Moreover, immunoprecipitation with the Flag antibody verified the binding of SET promoter 2 by the exogenous or overexpressed ZFX.

## 3. Discussion

In this study, we started with the deletion analysis to demonstrate the importance of the −157/+47 region for SET transcript 2 promoter activity in both HEK 293 and HeLa cells. Overexpression of potential transcription factors led to identification of ZFX as an activator of SET promoter. Mutagenesis analyses led to identification of the Site4 as the potent element accounting for much of the promoter capacity. This view was supported by the results of siRNA-mediated ZFX knockdown experiments. Chromatin immunoprecipitation experiments confirmed a direct interaction of ZFX with its cognate sequences. It is noteworthy that the activation followed a dose-dependent pattern, and the SET promoter was sensitive to either overexpression or downregulation of the ZFX level. Thus, proper SET expression appears to rely on an appropriate level of ZXF protein, and accurate bi-directional regulation may exist in cells.

Intriguingly, among the four ZFX-binding sites identified based on sequence analysis, only the one located closest to the transcription start site displayed activity, while the other three were largely dispensable. Such a phenomenon was observed previously in other genes. For example, human CD2AP gene promoter contains four canonical Sp1/3-binding sites, yet only the first and third sites contribute to the promoter activity [23]. We compared the four ZFX sites of SET promoter with the consensus ZFX-binding sequences (G)GGCCT [24] and found that actually Site3, not Site4, was the closest match. It should be pointed out that in this case the reported “consensus” sequences were empirical rather than laboratory-verified, and the affinity between ZFX protein and these putative binding sequences has not been experimentally measured or compared. It remains a question whether the specific sequence or the special location of Site4 may make it different from other sites. Future studies including in vitro binding experiments using purified ZFX, and swapping of the positions between Site4 and other sites in a reporter plasmid, may help to clarify this question.

The SET transcript 2 promoter is TATA-less and G/C-rich, and contains multiple Sp1 sites with high alignment score. However, reporter assay showed that Sp1 overexpression failed to impact the transcript 2 promoter activity. This is in a sharp contrast to the transcript 1 promoter that was reported to be activated by Sp1 in HEK 293, HeLa, and Acute Myeloid Leukemia (AML) cells HL-60 and HEL [21,22]. Such a discrepancy points to the distinct transcriptional regulation between the two SET transcripts. Alternatively, the lack of response by transcript 2 promoter to Sp1 overexpression may be attributed to a relatively high level of Sp1 that has saturated the SET promoter. Since we have focused on ZFX and did not perform Sp1 knockdown experiment, the possible role of Sp1 for transcript 2 promoter regulation should not be excluded.

ZFX contains a large acidic transcriptional activation domain, a nuclear localization sequence, and a C-terminal DNA-binding domain consisting of zinc fingers [25,26]. As a transcriptional activator, ZFX accounts for many genes’ expression in mouse embryonic stem cells (ESCs) [27]. ZFX could also transactivate c-Myc and the MHC class I HLA-A11 promoters in glioma stem cells (GSCs) and Leydig cells [28,29]. Besides, ZFX has been found to be overexpressed in several solid cancers, including breast cancer [30], prostate cancer [31], acute T-lymphoblastic and myeloid leukemia [32], hepatocellular carcinoma [33], and non-small cell lung carcinoma [34]. Revelation of ZFX-mediated transactivation of SET provides one mechanism by which ZFX may be implicated in multiple cell functions, e.g., those related to cancer development.

In summary, SET transcript 2 is a major transcription variant found in many cell types. We have characterized the SET transcript 2 promoter and demonstrated that the Zinc finger and X-linked factor ZFX transactivates the promoter through binding to a site closely located to the transcription start site. The pathophysiological significance of ZFX-mediated SET activation remains to be investigated. Similarly, the upstream signal that controls ZFX expression needs to be identified. Given extensive involvements of SET in Alzheimer's disease, PCOS, and cancers, identification of ZFX as a potent SET regulator is a meaningful step, and further investigations into the ZFX-SET signaling network may provide useful information for the development of new therapeutic modality against human diseases.

## 4. Materials and Methods

### 4.1. Cell Culture

HeLa and human embryonic kidney (HEK) 293 cells were purchased from the American Type Culture Collection (ATCC). Cell cultures were maintained in Dulbecco’s modified Eagle’s medium containing 10% fetal bovine serum (FBS, Invitrogen, Carlsbad, CA, USA). The medium was supplemented with penicillin and streptomycin (100 units/mL for each antibiotics). Cell cultures were incubated in a humidified incubator conditioned to 37 °C and 5% CO_2_ atmosphere.

### 4.2. Plasmid Constructs and Small Interfering RNA (siRNA)

The −855/+139 region containing the proximal promoter of human SET transcript 2 (NCBI Reference Sequence: NM_003011.3) was PCR-amplified with PrimeSTARE^®^ HS (Clontech, Mountain View, CA, USA) using the primers F1 and R, which were fixed with the NheI and XhoI sites, respectively (Table 1). The amplified fragment was cloned into the polycloning site preceding the luciferase coding sequences in pGL3-Basic plasmid (Promega, Madison, WI, USA), generating vector pGL3-855. Truncated SET promoters were produced by PCR using the combination of a common reverse primer (R, Table 1) with a specific forward primer (F2–F8, Table 1), and subcloned into pGL3-Basic. The ZFX site mutants at positions −146/−133, −99/−86, −93/−80, and −69/−56 were generated with primers Fr and Re (Table 1). Sequences of subcloned fragments were verified by DNA sequencing. The plasmids expressing various transcription factors (pcDNA3-E2F4, pcDNA3-E2F1, pcDNA3-EGR1, pCMV-Sp1, pCMV-E2F3a, or pCMV-E2F3b) were obtained from the nonprofit plasmid repository (Addgene, Cambridge, MA, USA). The pEF1-ZFX plasmid with the Flag epitope [28] was a generous gift from Shideng Bao, Lerner Research Institute, Cleveland Clinic, Cleveland, OH, USA.

Four ZFX-specific siRNAs and a RISC-free control siRNA were designed and synthesized by Dharmacon (Lafayette, CO, USA). The efficiency of ZFX-specific siRNAs for ZFX knockdown was assessed with real-time PCR and Western blotting. The designation and target sequences of ZFX-specific siRNAs are documented in Table 2.

### 4.3. Transient Transfection and Luciferase Assay

Transient transfection with plasmid DNA or siRNAs was carried out in HEK 293 and HeLa cells using the Effectene transfection kit (Qiagen, Valencia, CA, USA) and the HiPerFect transfection kit (Qiagen, Valencia, CA, USA), respectively. Cells were seeded in 96-well plates with 100 μL of antibiotics-free medium 24 h before transfection. Following 7 h exposure to transfection reagents, the medium was changed to a regular one. At 24 h post-transfection, cells were lysed and luciferase activity was measured using the Dual Reporter assay system (Promega, Madison, WI, USA) and the LB960 microplate luminometer. The pGL3-Basic vector was used as a control, while the pRL-TK (Promega, Madison, WI, USA) plasmid expressing the renilla luciferase was used as an internal reference to standardize the luciferase activity. For dose-response experiment, 0–100 ng of ZFX expressing plasmid (pEF1-ZFX) and 100 ng of promoter reporter plasmid/2.5 ng of pRL-TK plasmid were co-transfected into HeLa cells. Transfection experiments were performed in triplicates. At least three independent experiments were repeated.

### 4.4. Chromatin Immunoprecipitation (ChIP) Assay

CHIP assay was performed using the EZ-CHIP kit (Millipore, Billerica, MA, USA). Briefly, approximately 1 × 10^7^ cells were fixed with 1% formaldehyde and sonicated on ice with 15 s bursts that were repeated 15 times with 15 s intervals. Optimization experiments were performed in order to obtain an optimal length of chromatin fragments with 200–1000 bp of genomic DNA (Figure S3). Target chromatin fragments were enriched with 2 µg anti-ZFX antibody (Cell Signaling, Beverly, MA, USA) and immunoprecipitated with protein G agarose beads. In a parallel experiment, non-immunized rabbit IgG was used as a negative control. In ZFX overexpression experiments, 2 µg anti-FLAG M2 antibody (Sigma, Saint Louis, MO, USA) was applied as a verification approach. After extensive washing of beads, DNA was freed following protease K digestion. DNA was purified and analyzed using PCR with primers encompassing the putative ZFX-binding sites of SET promoter (Table 1). To assess ZFX binding to SET promoter sequences in the luciferase reporter plasmid, PCR was performed using a forward primer targeting the SET promoter sequences and a reverse primer targeting the vector sequences. Following gel electrophoresis, DNA bands were quantified with densitometry analysis.

### 4.5. RNA Extraction and Real-Time Quantitative-PCR

HeLa cells were transfected with either ZFX siRNA or siRNA control using the RNeasy Plus Mini Kit (Qiagen, Valencia, CA, USA). At 48 h post-transfection, total RNA was isolated from cells and quantified with NanoDrop 1000 spectrophotometry (Thermo Scientific, Rockford, IL, USA). 1 μg of total RNA was reverse-transcribed in a 20 µL reaction using the High Capacity RNA-to-cDNA Kit (Applied Biosystems, Carlsbad, CA, USA) following the manufacturer’s instructions. The resulting cDNA was subsequently amplified using primers P1 to P4 (Table 1) and the SYBR Green PCR Master Mix (Applied Biosystems, Carlsbad, CA, USA). GAPDH was employed as an internal reference to normalize the PCR results. Final products of real-time PCR were analyzed on 1.5% agarose gel, and a single DNA band pattern with the predicted size confirmed the accomplishment of specific amplification (Figure S4). The ZFX mRNA levels were calculated according to the 2^-ΔΔ*C*t^ method. The threshold cycles were determined in triplicates with the use of 7900 Real-Time PCR System (Applied Biosystems, Carlsbad, CA, USA). 

### 4.6. Western Blotting Analysis

At 72 h post-transfection, cellular proteins were extracted from HeLa cells with RIPA buffer (Boston BioProducts, Boston, MA, USA) containing 5 mM sodium fluoride (NaF), 1 mM sodium vanadate (Na_3_VO_4_), and 1 mM phenylmethylsulfonyl fluoride (PMSF), supplemented with 1% Protease Inhibitor Cocktail (100X, Thermo Scientific, Rockford, IL, USA). Protein concentration was measured with the Bradford Assay. 50 µg of protein was loaded and separated in an 8% polyacrylamide SDS-gel and electrotransferred onto polyvinylidene fluoride membranes. The membranes were incubated with primary antibodies, followed by incubation with matching HRP-conjugated secondary antibodies. Color development was carried out using the ECL reagents (Pierce, Rockford, IL, USA). GAPDH was detected and the results were used as protein loading controls. The primary antibodies used were: anti-ZFX (1:1000, Cell Signaling, Beverly, MA, USA) and anti-GAPDH (1:1000, Santa Cruz, TX, USA).

### 4.7. Statistical Analysis

Averages and standard deviations (SD) were calculated for each experimental group. All statistical analyses were performed with the use of SPSS 13.0 Software (SPSS, Chicago, IL, USA). One-way analysis of variance (ANOVA) was used to evaluate quantitative data containing more than two groups. Data that passed ANOVA test were further analyzed using the student’s *t* test for one-to-one comparison. Statistical significance was set at the level of *p* < 0.05.

## Figures and Tables

**Table 1:** Sequences of primers used for DNA cloning, nucleotides mutation, ChIP assay, and real-time PCR.

Designation	Primer	Sequence	Size (bp)
P1 (pGL3-855)	F1	TCTTACGCGTGCTAGCAGTAGAACTTGTTGGCCCCT	994
P2 (pGL3-654)	F2	TCTTACGCGTGCTAGCCTTCAGTGTCGTAACCTTTA	794
P3 (pGL3-327)	F3	TCTTACGCGTGCTAGCGCAGGGAAGCCGCTCGCTCA	467
P4 (pGL3-251)	F4	TCTTACGCGTGCTAGCGCCTGCCGCGCGCCAGTGTC	391
P5 (pGL3-157)	F5	TCTTACGCGTGCTAGCTCCTCCCACCTCTCGCCGGG	297
P6 (pGL3-85)	F6	TCTTACGCGTGCTAGCTGGCGCCGCGGCGCGAGCCT	225
P7 (pGL3-45)	F7	TCTTACGCGTGCTAGCTCCGCGCGGGGCCTGGCGCGCCT	185
P8 (pGL3+47)	F8	TCTTACGCGTGCTAGCGCTGGCTGGATCGCCGAGCG	93
P1–P8	R	GATCGCAGATCTCGAGGCCCCGGCCCGGGCTCCTGT	
M1	Fr1	TCTTACGCGTGCTAGCTCCTCCCACCTAACGCTTAGACATCTGTCTCCGCGCCGCGACGAT	329
	Re1	CGCAGATCTCGAGGCCCCGGCCCGGGCTC	
M2	Fr2	TCTTACGCGTGCTAGCTCCTCCCACCTCTC	101
	Re2	CGCCGCGGCGCCAGATGTCTAAGCGTTGGGATGAGGGGGCGGAGTC	
	Fr2′	AGACATCTGGCGCCGCGGCGCGAGCCTC	249
	Re2′	CCGCGACGATCGCAGATCTCGAGGCCCCGGCCCGGGCTC	
M3	Fr3	TCTTACGCGTGCTAGCTCCTCCCACCTCTC	119
	Re3	TGATGTCTAAGCGTTTCGCGCCGCGGCGCCATTGGC	
	Fr3′	CGCGAAACGCTTAGACATCAGGCGGAGCATCCGCGCG	230
	Re3′	CCGCGACGATCGCAGATCTCGAGGCCCCGGCCCGGGCTC	
M4	Fr4	TCTTACGCGTGCTAGCTCCTCCCACCTCTC	146
	Re4	CGCGATGTCTAAGCGTTATGCTCCGCCTCCGGCC	
	Fr4′	CATAACGCTTAGACATCGCGCGCCTGCGCCCTGCG	203
	Re4′	CCGCGACGATCGCAGATCTCGAGGCCCCGGCCCGGGCTC	
ChIP-promoter	F-pr	TCTCGCCGGGCCCTGTGTCT	173
	R-pr	CGCCTCCTCCTCCACCTCCT	
ChIP-plasmid	F′-pd	CGCCCAGGCCAATGG	322
	R′-pd	TTGGCGTCTTCCATGGTG	
ZFX	P1	TTGCTGAAATCGCTGACGAAG	133
	P2	GCAATCGGCATGAAGGTTTTGAT	
GAPDH	P3	ACCATCTTCCAGGAGCGAGA	71
	P4	GACTCCACGACGTACTCAGC	

**Table 2:** Designation and sequences of siRNAs used for ZFX knockdown.

Designation	Target Sequence
si-ZFX1^#^	UGAAAUCGCUGACGAAUGG
si-ZFX2^#^	AAGCAGAAAUUGUCACUGA
si-ZFX3^#^	GGACGUUGUUAUAGAAGAU
si-ZFX4^#^	ACACAGAGUCGGAAAUUGA

## Supplementary Materials

Supplementary materials can be found at www.mdpi.com/1422-0067/17/10/1737/s1.

## Abbreviations

ZFX	Zinc finger and X-linked factor
PCOS	Polycystic Ovary Syndrome
TAF-1β	Template Activating Factor-1β
I2PP2A	protein phosphatase 2A inhibitor
AD	Alzheimer’s disease
ChIP	Chromatin immunoprecipitation
AML	Acute Myeloid Leukemia
