# Supplementary material for: Zinc Finger and X-Linked Factor (ZFX) Binds to Human SET Transcript 2 Promoter and Transactivates SET Expression
Source: Int J Mol Sci. 2016 Oct 20;17(10):1737. doi: 10.3390/ijms17101737 (PMC5085766; doi:10.3390/ijms17101737)
Supplement: Supplementary file 1 [file ijms-17-01737-s001.pdf]

Supplementary Materials: Zinc Finger and X-Linked Factor (ZFX) Binds to Human SET Transcript 2 Promoter and Transactivates SET Expression

Siliang Xu, Ping Duan, Jinping Li, Tristan Senkowski, Fengbiao Guo, Haibin Chen, Alberto Romero, Yugui Cui, Jiayin Liu and Shi-Wen Jiang

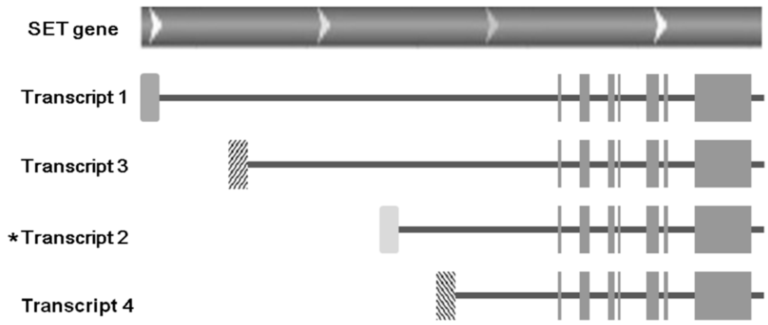

Figure S1. SET transcript variants produced by alternative promoters. *SET* gene is 19,742 bp long and located at human chromosome 9q34. It generates four transcription variants with transcript 1 and 2 being the two major transcripts. Gray boxes represent exons. Each SET variant consists of 8 exons. Note the divergent exon 1 among the transcript variants. *, the transcript which we focused on in this study.

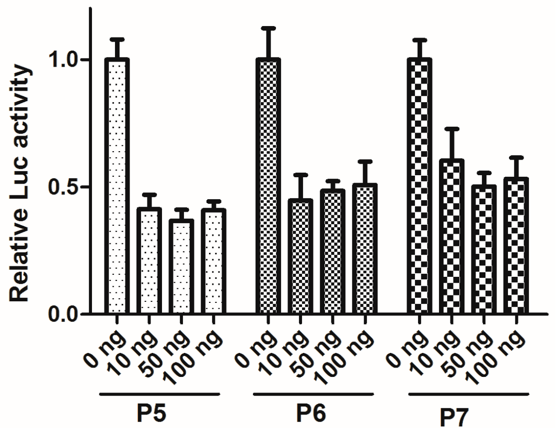

Figure S2. E2F3a overexpression displayed a similar inhibitory activity in P5, P6 and P7 promoters. HeLa cells were co-transfected with increasing amounts of pCMV-E2F3a (0–100 ng) plasmid DNA, pCMV vector DNA as "stuffer" to keep a constant DNA amount, and 100 ng of P5, P6 or P7 reporter plasmids. Luciferase activity was measured at 24 h post-transfection. A uniform inhibition was observed in all the three promoter constructs tested, suggesting either a non-specific nature of the effect or the presence of a negative element(s) further downstream of the deleted region. Quantitative data is presented as means ± SD from three independent experiments.

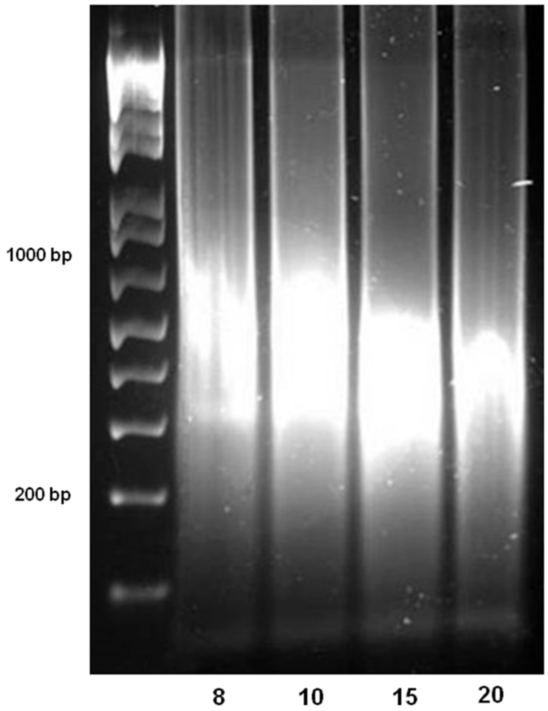

Figure S3. Optimization of sonication. Increasing number of bursts, ranging from 8 to 20, was applied. Following sonication, DNA was freed from chromatin, isolated, and resolved in agarose gel (2%) electrophoresis. Numbers at the bottom show the sonication times. 15 times of 15 s sonication bursts with 15 s intervals is optimal for achieving 200–1000 bp lengths of DNA fragments, an ideal length range for ChIP assay.

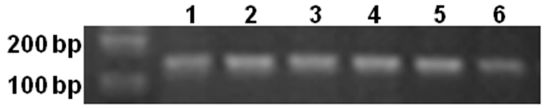

Figure S4. Specificity of real-time PCR used for the measurement of ZFX expression level. Final real-time PCR products (35 cycles) were resolved by electrophoresis using 1.5% agarose gel. Left lane is the DNA markers. Lanes 1–3: Real-time PCR products of triplicate experiments in which Hela cells were transfected with pEF1-vector; Lanes 4–6: Real-time PCR products of triplicate ZFX overexpression group transfected with pEF1-ZFX. The clear, single DNA band with predicted size of 133 bp indicated a high PCR specificity has been achieved, and the CT values accurately measured the ZFX transcript levels.
